# Intergenerational Effects on the Impacts of Technology Use in Later Life: Insights from an International, Multi-Site Study

**DOI:** 10.3390/ijerph17165711

**Published:** 2020-08-07

**Authors:** Shannon Freeman, Hannah R. Marston, Janna Olynick, Charles Musselwhite, Cory Kulczycki, Rebecca Genoe, Beibei Xiong

**Affiliations:** 1 School of Nursing, University of Northern British Columbia, Prince George, BC V2N 4Z9, Canada; 2 Health and Wellbeing Strategic Research Area, School of Health, Wellbeing and Social Care, The Open University, Milton Keynes MK7 6AA, UK; Hannah.Marston@open.ac.uk; 3 School of Nursing and Department of Psychology, University of Northern British Columbia, Prince George, BC V2N 4Z9, Canada; Janna.Olynick@unbc.ca; 4 Centre for Innovative Ageing, Swansea University, Swansea SA2 8PP, UK; c.b.a.musselwhite@swansea.ac.uk; 5 Faculty of Kinesiology and Health Studies, University of Regina, Regina, SK S4S 0A2, Canada; cory.kulczycki@uregina.ca (C.K.); rebecca.genoe@uregina.ca (R.G.); 6 School of Health Sciences, University of Northern British Columbia, Prince George, BC V2N 4Z9, Canada; bxiong@unbc.ca

**Keywords:** digital, intergenerational communication, gerontology, aging, family, cross-cultural research, qualitative research

## Abstract

As the use of technology becomes further integrated into the daily lives of all persons, including older adults, it is important to investigate how the perceptions and use of technology intersect with intergenerational relationships. Based on the international multi-centered study Technology In Later Life (TILL), this paper emphasizes the perceptions of older adults and the interconnection between technology and intergenerational relationships are integral to social connectedness with others. Participants from rural and urban sites in Canada and the UK (*n* = 37) completed an online survey and attended a focus group. Descriptive and thematic analyses suggest that older adults are not technologically adverse and leverage intergenerational relationships with family and friends to adjust to new technologies and to remain connected to adult children and grandchildren, especially when there is high geographic separation between them. Participants referenced younger family members as having introduced them to, and having taught them how to use, technologies such as digital devices, computers, and social networking sites. The intergenerational support in the adoption of new technologies has important implications for helping older persons to remain independent and to age in place, in both age-friendly cities and in rural communities. The findings contribute to the growing literature in the fields of gerontology and gerontechnology on intergenerational influences and the impacts of technology use in later life and suggest the flexibility and willingness of older persons to adopt to new technologies as well as the value of intergenerational relationships for overcoming barriers to technology adoption.

## 1. Background

From monitoring personal health and wearable devices to playing online games and using social media to connect with friends and family, technology has become a valued component of daily life for many individuals. Interest in technology has steadily increased over the past decade, associated with unprecedented growth and innovation in information and communication technologies (ICTs) [1,2]. There has been an increase in the proportion of older adults (persons aged over 65 years) in countries across the world utilizing technology [3]. As the use of technology and associated ICTs increases, there is a greater need to expand the understanding of the intersection of technology, ageing, and intergenerational relationships. A particular gap in knowledge exists regarding the role of intergenerational elements in motivating older adults to learn how to use technology and associated ICTs. 

Across the globe, societies are aging rapidly due to increased life expectancy as a result of better health and social care, and lower birth rates [4]. Recent United Kingdom (UK) population estimations suggest the proportion of those aged 65+ years in rural and urban environments will increase by 50% between 2016 and 2039, whilst those aged <65 years are projected to increase by eight percent in urban areas and to stagnate in rural locations [5]. In 2014, 15.6% of the Canadian population, equating to over 6 million persons, were aged 65, and it is predicted that by 2030, older adults will exceed 9.5 million persons, accounting for 23% of the Canadian population [6]. In Canada, the majority of older adults (56.4%) lived with a spouse or a common-law partner in 2011 while about one-quarter (24.6%) lived alone [6,7].

The increase in the migration of younger cohorts from rural to urban areas and of older adults from urban to rural areas leaves an increased proportion of older adults in rural areas who prefer to “age in place” [8,9]. Research focused on aging in urban areas has emphasized the challenges older adults face in accessibility, especially in access to public transportation, shopping, and green space [10]. As geographic separation between family members increases, the role of ICTs in helping to strengthen and maintain family bonds becomes more important [11]. However, the extent to which older adults use technologies for this purpose remains unclear. Although, in the future, aged cohorts may be more “tech savvy” [12], having used technologies regularly across their life course, new technologies may still arrive that could be disproportionately challenging for older people to adopt.

Technology (e.g., digital devices, the Internet, digital gaming, and mobile apps) use in later life is a growing field of research, with much new exploration and study [13,14,15,16]. Technology and associated ICTs are often aimed towards improving the health, wellbeing and quality of life of older adults, whether through applications for home healthcare and connected health services [17], medication reminders [18,19], mirrors that display health data [20], or wearable technology [21]. Technology use to enhance communication is routine practice for many older adults, with home computers being used to create a common interest among older and younger family members and improve family ties [22].

Technology use among older adults is growing [3]. For example, in Canada, between 2007 and 2016, Internet use increased from 32% to 68% among those aged 65 and older [23]. In 2016, 85% of people aged 65–69 used the Internet compared to 62% of those aged 70–79 and 40% of those aged 80 years and older [23]. Challenges with technology have been linked to age, evidenced by differences in use [24,25] and variation in the learning of technology (computers and Internet) between older and younger adults [12,26,27,28,29,30,31]. Older adults in Canada were less likely than younger adults to perceive technology as useful for communicating with others, making informed decisions, and saving time [23]. Several studies reported that Internet use is lower among older-aged cohorts than younger cohorts [32,33,34]; however, there is evidence of a cohort effect as there has been an increase in technology use within older-aged cohorts over time [2]. Older adults who do use the Internet report lower confidence in their ability to do so than younger adults [35], which may be tied to challenges older adults experience with technology use (e.g., visual difficulties and cognitive declines) [36,37]. Older adults are likely to make more errors and require assistance when learning computer systems and software [36,37].

Previous research suggests that older adults may be “technophobic” [38,39] and struggle to use technology [2], as they embrace technology differently and at a slower pace than younger adults [2], [32,40]. As the Canadian and UK populations age, differences in technology adoption and use across age cohorts may increase, amplifying the “generational gap” [41]. While learning to use technology serves as a rite of passage for today’s youth, playing an important role in the self-definition of young adults [42], this may not be the case for older generations. Individuals not born into the current rapidly evolving digital age, sometimes referred to as “digital immigrants”, must find ways to adapt to a changing society [43]. Rama noted that each “technology generation” may have been affected by common experiences during their formative years that influence behaviours towards and the use of technology [44]. However, these notions are challenged by Bennett and Maton, who note the diverse range of experience and engagement with technology among youth, as well as by Loos, who describes technology use as a spectrum affected not only by life stage but also by socialization and degree of age-related functionality [16,45]. Technology use is complex and can no longer simply be split into user vs. non-user groups. Instead, the heterogeneity in the use of technology includes not only use of the technology for an intended purpose but also the meaning and value that the use of technologies has in mediating social relationships and connection to the external world [46]. Existing research highlights differences in technology use between the generations; however, research on the connection between intergenerational factors, social variables, and technology use among older adults is less prevalent, with notable exceptions including [26,27,28]. However, other research suggests that age is not a consistent driving factor associated with aversion to technology such as computer anxiety [24]. As such, it remains less clear how factors such as intergenerational intelligence, solidarity, and adaptiveness apply to the learning and use of technology, especially by older adults [47,48,49,50,51].

Younger generations are the dominant early users and adopters of social networking sites [12,52], with few older adults (between 10% and 27%) using this form of technology [2,3]. Social networking and other technologies present opportunities for older generations to connect with younger generations and individuals in diverse geographic locations [22,53,54,55]. Technology has been shown to enhance an older adult’s quality of aging [56], independence [57], social status [56], interpersonal relationships, control, self-esteem, and integration into society [57,58]. To understand how to meet the needs of an aging population in a technology-suffused society, it is useful to understand why older adults choose (or not) to use technology and whether (or not) they perceive the reasons driving their choice as constraints requiring negotiation or benefits to everyday life. The challenges to acquiring new technology skills and strategies for connecting with younger generations to overcome them suggest the importance of intergenerational influences on older adults’ understanding and use of technology, which must be further explored.

The above findings are concerning in light of research reports that older adults are more likely to experience loneliness and isolation [34]. There is, to date, a growing body of scholarly work exploring the relationships between intergenerational relationships and technology [27,28], offering insight into how technology and associated ICTs lay within and across intergenerational networks. Taipale and colleagues [27] discuss ICT use through various lenses including both older and younger adults—a generational perspective, the family, and the home. To further extend research in this area, we describe further the relationship between technology use and interpersonal relationships—more specifically, the how older adults’ understanding and use of technology is affected by their intergenerational relationships.

## 2. Methods

### 2.1. Aims and Objectives

The Technology In Later Life (TILL) study examined the experiences of older adults aged 70+ years with technology, exploring how they adopted, accepted, and used various types of technology. Subsequently, the team sought to identify the implications of using ICTs for current and future aging populations in rural and urban locations.

### 2.2. Study Location

The Technology In Later Life (TILL) study was an exploratory study conducted in Canada and the UK across four study sites. Canada and the UK were selected for this study as they both have aging populations and exposure to technology and contain different rural and urban populations. In each country, two sites were selected: one rural and one urban. The rural site in Canada was the town of McBride (BC), and the urban site selected was the city of Regina (SK). The rural sites in the UK included the village of Cwmtwrch and the village of Ystalyfera in Wales, and the urban site was the town of Milton Keynes (Buckinghamshire) in England.

### 2.3. Procedure

Participants were recruited through the use of posters and mailing list scripts tailored to each site distributed to local organizations including the Older People’s Forum, seniors’ centers, public libraries, seniors’ community newsletters, and local public radio. Participants were also recruited through word of mouth in the community. Participants each voluntarily contacted the lead investigator for the research site closest to them to request to participate in the study. Upon contact, the participants were sent an email containing a link to the online survey, information on the study and a request for written consent to participate, and an invitation to set a date to join a focus group interview.

All participants completed the online survey prior to participation in a focus group. The survey was an iteration of an earlier survey [14,59], which covered eight domains: (1) technology use, (2) internet ownership and use, (3) social networking, (4) digital device ownership, (5) purchasing patterns, (6) quantified self- and life-logging, (7) information sharing and privacy issues, and (8) demographics. Bivariate analyses of the survey data were conducted using SPSS version 24. An inductive approach was taken to generate new knowledge from the qualitative data. A descriptive approach is beneficial for an initial study, as such an approach allows the researchers to richly describe the phenomenon being studied.

Focus group discussions, led by the lead researcher from each site, lasting between 40 and 60 minutes, were digitally audio-recorded and then transcribed verbatim in Microsoft Word by a UK-based transcription company. All the lead researchers were experienced in conducting qualitative research analyses and in leading focus groups. A semi-structured interview guide containing questions and probes was used to facilitate discussion (Supplementary Materials). The questions examined several areas including the ownership of technology, the purpose for using technology, internet social media use, life-logging, privacy issues and the sharing of information (e.g., what type of information and rationale for sharing), and willingness to embrace new technology (Supplementary Materials).

Content and inductive analyses [60] were conducted across all the transcripts. Given the exploratory nature of this analysis, the transcriptions were read closely for familiarization with the data, coded, and analyzed thematically. The data were classified into categories as a way of describing key themes [61]. In addition, areas of concordance and discordance were examined through the analysis. Specifically, open coding, with the creation of categories and abstraction, was undertaken. Coding was first conducted independently by a research assistant, trained in qualitative research methodologies and experienced in conducting analysis, and by a co-investigator, both of whom then came together to come to a consensus on the coding. Discrepancies were addressed by recoding areas of discordance, and then, the transcripts were reanalyzed by the research assistant and reviewed by a co-investigator of the study to promote accuracy and trustworthiness [62]. Ethics approval was granted by all four institutions.

## 3. Findings

Thirty-seven participants both completed an online questionnaire and attended a focus group discussion. This included 20 rural participants (McBride, Canada, *n* = 10, Cwmtwrch and Ystalyfera, UK, *n* = 10) and 17 urban participants (Regina, Canada, *n* = 6 and Milton Keynes, UK, *n* = 11) from 2015 to 2016. Most participants were female (67.6%), retired/not employed (86.5%), and in their late 70s (mean age, 77.4 years). Five themes were identified relating to intergenerational relationships. Three themes focused on the benefits of intergenerational relationships to support use of technology including 1) Motivation for older adults to use technology, 2) Use of technology as a facilitator of intergenerational connection and 3) Technology use for safety reasons. Additionally, two themes focused on the impediments of intergenerational relationships to use of technology including 1) Using technology to appease younger family members; and 2) Learning how to use technology in later life.

All participants used technology, the majority of whom did so on a regular basis (Table 1). Nearly all participants used a computer (97.3%) and owned a computer (89.2%). Most participants had used a computer for at least 10 years (75.7%) and used a computer more than once per day (62.2%). All participants used a digital device, typically a mobile/cell phone (70.3%), and to share information (82.7%). Nearly all participants identified having internet at home (94.3%) and most had used the internet for more than 10 years (75.8%). Participants used technology for a variety of tasks including e-mail, word processing, playing games, making telephone calls, online shopping, online banking, sharing information, social networking, searching/checking information, instant messaging, reading, uploading content, and lifelogging. Over half reported using social media (54.1%, *n* = 20) with more Canadian participants’ self-reporting use of social media when compared to participants from the UK (62.5% vs. 47.6%) (Table 2).

### 3.1. Benefits of Technology Use—Motivation for Older Adults’ Use of Technology

A primary motivation for participants to use technology was as a “digital gathering place” to communicate with family, especially adult children and grandchildren, and friends. Participants communicated through technology in a variety of ways including Skype, Facetime, e-mail, social networking sites (e.g., Facebook), and texting through cellular networks or WhatsApp. Interestingly, it was common that participants who used technology were taught how to do so by younger family members. The value of digital communication was enhanced when participants’ children and/or grandchildren lived far away.

“Skype is brilliant. I’ve got a daughter in Spain, I’ve got a granddaughter in Spain, I’ve got a son in the West Indies and a daughter in London, and Skype is one of the most brilliant things that’s happened because you can see, you can talk.” [MK6, male].

“I’ve used Skype because my daughter lives in South Africa, but it’s an atrocious service because South African broadband is atrocious. We now use Apple FaceTime and that is far superior.” [MK3, male].

Given the time difference across geographic distances, technology afforded both parties the flexibility to schedule face-to-face communication at a convenient time. Social networking platforms including “Facetime with other members of the family” [Wales1, female] as well as e-mail, Facebook, and WhatsApp were used to engage with family to “…keep track of the grandkids and great grandkids” [McB4, male]. Participants connected across the generations as noted by one participant who shared, “I go on Facebook and I go on Skype with my daughter in Australia and I do research things. Last night I was talking to my grandson, who’s seven” [MK2, female]. It is also useful to note that participants adjusted the platforms they used not only due to personal preferences but also in response to the variance in the infrastructure and broadband support across the locations.

### 3.2. Benefits of Technology Use—Technology as a Facilitator of Intergenerational Connection

Older adults reported using technology to connect with friends and family members, and to share information, also likely with family members. Participants often used computers for email (85.3%) and social networking (38.2%), most often in their own home (97.1%) and occasionally at an adult child’s home (17.1%). Social networking sites were used to stay connected with children and/or grandchildren and friends, to share photos and information with friends and/or family, and to keep up to date with news. The Internet was used for sending/receiving e-mails, social media, making phone calls through Skype/Viber, and instant messaging. Older adults both created and sent content (e.g., photos and emails), as well as receiving content. It was both older adults and their family members/friends who took turns initiating contact.

Most participants identified that they used technology to write or speak with other family members; there were a few instances where participants reported using technology to partake in and share the hobbies of younger family members. Older adults were keen to try new things with their grandchildren such as interactive videogames and immersed themselves in the flow of the games. One participant noted, “[…] Jumping up and down to the things that they’ve got on the screen when you play tennis or jump up and down and dance, or whatever you’re chasing, something. Yes. Video games, I suppose. Childish ones.” [McB2, female]. Another participant used her daughter and granddaughter’s iPad to take pictures of the community garden. Participants suggested that technology is not only used to connect and communicate with younger family members but also to learn about and actively participate in activities with younger generations.

### 3.3. Benefits of Technology Use—Technology Use for Safety Reasons

Of the participants using technology to stay in contact with family, some also acknowledged having started using a digital device for safety reasons at the suggestion of another family member, commonly an adult child. Most participants reported owning a mobile device or cell phone, many of whom owned these devices for “safety” [Regina2, Female] and “emergencies only” [MK5, female]. One participant living in rural British Columbia described how they started using a digital device specifically for driving purposes as well as feeling the need to maintain a sense of peace with their adult children.

“I got the cell phone because my kids kept thinking something was going to happen to me. I said, “Well you know if I have a breakdown on the highway, we managed for 70 years for God’s sake by just stopping someone and they’d help you. But now, “Oh my God they could murder you.” So, this was supposed to be a safety element to keep peace in the family.” [McB1, female].

This participant further described displeasure with the cell phone because it cost them money each month and they never used the device. Several participants identified that they got digital devices at the suggestion of an adult child after having suffered a health scare. For example, when asked why they got a cell phone, one participant replied, “Oh, well it was the bright idea of my son. I had a mini stroke… ever since, but they’re [kids] always frightened… of a recurrence. So, my son gave me a cell phone, his old one, which I used right away, or more or less. I think, they decided that I should have one, because I did get a few dizzy spells. So, now I just use it” [McB2, female].

Even though it was often a younger family member, such as an adult child, who suggested the participant carry a digital device for safety-related reasons, most participants had positive perceptions of using technology for such reasons. For example, one participant spoke positively of how they wore a certain piece of technology that they can press in an emergency situation to notify a family member or emergency service that help is needed. While it seems that most participants use technology to keep in touch with younger family members, the reasons for this contact vary, from safety and emergency situations to routine check-ins with children and grandchildren.

### 3.4. Impediments to Technology Use—Using Technology to Appease Younger Family Members

In some instances, participants seemed to use technology to make a younger relative happy even if they did not seem to need the technology. For example, “I don’t even have an iPhone or iPad so I’m really out of date…I will get more modernized so that my children will be happy” [Regina2, Female]. Another participant stated, “I’ve got a tablet that I was to take away with me because my grandchildren said it would be useful to have and I wouldn’t be using theirs whenever I’m away on holiday with them. I don’t get on terribly well with a tablet…” [MK2, female].

Common responses for why participants owned technology included similar motivations, stemming from the children: “…the kids decided we should have one [computer]” [McB4, male] and that their grandchildren were putting pressure on them to keep up with the latest technology. Furthermore, one participant explained that they were learning technology because the “…grandchildren push me and they go, ‘Oh Nana, you’re so far behind, you should be up to date and you should be doing this and doing that.’ So, they want me to be up to date with all the latest technology and I’m not.” [Regina3, female].

In certain cases, younger family members purchased technology for older family members as gifts. One participant reflected on a life logging device they owned, explaining, “My daughter bought it for my birthday…” [Wales1, female] after her husband began experiencing a health decline. These examples illustrate, across the different study sites, how the respective participants felt about technology and how digital devices had been implemented into their lives without consideration of their respective feelings, needs, and choice.

### 3.5. Impediments to Technology Use—Learning How to Use Technology in Later-Life

Many participants used computers as integral components of their jobs decades ago and were among the early adopters of computing technologies. One participant who was familiar with computers explained that they used to do IT at Milton Keynes College. Similarly, a participant from McBride learned the fundamentals of using a computer for their accounting position, explaining that they learned about spreadsheets. However, with the rapid pace of technology development, the technological skills participants had employed prior to retirement became quickly outdated. 

Participants described that the challenges in keeping up with the rapid pace of changes in the technology itself were compounded by their frustrations in keeping up to date on the expanded language used to describe the technologies. Participants described the complexity in language and terminology used in technology tutorial classes and instruction manuals as too complicated and inhibiting their ability to adopt new technologies. One participant identified that instructors at computer classes “go way too fast for me. I can’t keep up; there is too much new information… the language like computer and technological language is totally different from what we were raised with” [McB2, male]. Another participant identified similar grievances about learning to use technology, such as the fact that they “can’t understand technology words” [McB1, female] in instruction manuals and that when speaking with information technology (IT) specialists, the IT specialist would explain too quickly. 

Although participants noted how they were confused about how to use technology, they still managed to do so, most commonly with assistance from younger family members. Participants were frequently introduced to digital devices and to social networking sites by a relative or adult child. Participants alluded to younger family members playing a key role in the learning process, saying things such as “My son set it [Skype] up…” [MK2, female] and “Oh, my daughter is the one that does all the computerizing. She helps…” [McB2, female]. They emphasized that they were not technophobic or averse to use of the technology itself but felt outpaced by the speed of change of technology. For many, they were unable to overcome the language barriers created to adapt and adjust to changes in technology on their own or with those of a similar age. Instead, they would connect with younger generations for help. Where confusion over technology existed, younger family members took on a teaching role, especially for newer technologies such as digital devices and social networking programs. “I ask my grandchildren. ‘Okay, how do I do this?’ They say, ‘Don’t you know?’ But they will help me eventually” [Regina3, female]. Younger generations were able to bridge the technology gap and communicate complex language in lay language that was non-threatening. “Anything I want to know, I have to phone up my sons or my grandchildren because they’re a lot more knowledgeable than I am…” [MK1, female]. 

Even after being introduced to technology and learning how to use it, participants continued to contact their adult children and other relatives for assistance when faced with difficulties. For instance, one participant stated that “My son is an IT expert. If I have any problems, ‘Can I speak to the IT man please.’ He knows it’s me. He sorts my problems” [MK3, female]. Some participants seemed to solely rely on younger family members for information when necessary. For instance, one participant concluded, “If I need to know something, I will get my daughter to look it up on her, whatever thing she packs in her pocket” [McB4, male].

## 4. Discussion

For many older adults, intergenerational relationships are leveraged to support the understanding and use of technology. The challenges in the adoption of and adaptation to the rapid developments in digital technologies facilitate opportunities and meaningful purposes for participants to connect and communicate with younger generations. The leveraging of technologies, including social media and virtual communication platforms, supported older adults in maintaining and enhancing social connections, especially with adult children and grandchildren who lived in different cities and countries. These findings support the idea that the use of digital technologies can enhance social connectedness across generations; as Taipale noted, “[…] distributed families can today nevertheless remain connected and feel a sense of togetherness, even when their members are not physically close to one another” [28].

The benefits of intergenerational relationships for technology, including motivation for older adults’ use of technology and the use of technology as a facilitator of intergenerational connections, underlie each domain of the WHO Checklist of Essential Features of Age-Friendly Cities [63]. Furthermore, this reinforces the need for a revised smart age-friendly ecosystem framework as coined and posited by Marston et al. [10], who proposed an extension, noting that these features also apply to the rural, and non-urban, context. The desire to mitigate the digital divide fuels older adults’ motivation to invest time in building and fostering intergenerational digital connections. Previous research similarly suggests that computers are commonly used by older adults as a method of communication with younger generations, serving as a gateway to the world of younger family members and a means to strengthen relationships [64]. Studies show that individuals will often play games, not because of enjoyment of the game itself, but because of the social interaction with others with whom they are playing [65]. Therefore, when creating an age-friendly environment or helping older persons to age in place, it is worthwhile to challenge those designing built environments to consciously address how they may seize opportunities to effectively and efficiently leverage ICTs to facilitate intergenerational engagement. 

Older adults leveraged technology to connect, communicate, and actively participate in the interests and hobbies of their adult children and grandchildren in online formats, including digital gaming and photography. Participants encouraged and enjoyed interacting with younger family members to learn about different technologies (e.g., digital games) as a way of immersing themselves in the culture of younger generations. As previous research illustrates, participants in this study were using digital games as a “computational meeting place” that supported meaningful social interactions and shared motivation for group gaming [66]. Further evidence shows that gaming technologies foster intergenerational group interactions of up to four generations, including adult children and extended families [67]. Our study revealed findings similar to those noted above but for multiple digital technologies, which suggests a more universal and generalizable use of technologies among older adults to increase intergenerational family social interactions as a “digital gathering place”. Health limitations, the costs of transportation, and social isolation can create barriers for travel, all of which might explain why communication technologies such as Skype were often used to connect with family members. These technologies can come close to replicating the face-to-face experience of conversing with another person and are an effective communication method to use when travel is not an option. The extended value of the support of intergenerational connection may be further amplified given the context of COVID-19 and in the post-COVID-19 context.

Language and terminology often impede the ability of older adults to learn how to use technology. This disconnect and incomplete understanding of technological language could explain why few respondents identified using social media/networking sites but went on to further indicate they do in fact use this form of technology. This discrepancy in responses may stem from a lack of clarity in the question about what social networking entails for the respective participants, or this may reflect a lack of recognition by older adults that they did in fact use social media/networking platforms. Despite these complications, participants were able to use technology and associated ICTs by learning to do so with their adult children and grandchildren, who were able to translate the jargon and technical terms used in information technology courses into a language that older adults could understand within the context of intergenerational relations. This is consistent with the findings from previous studies showing that adult children often initiate the technology use process for older adults and that extended family members (such as grandchildren) are important educators for older adults as they learn to use technology [64,65,66,67,68,69].

Intergenerational informal education between those with existing relationships may be more effective for knowledge/information exchange. When considering why adult children and grandchildren were common educators, there are a few ways to explain this finding. First, older adults might feel more comfortable learning from family members due to feelings of trust. Second, as it was often adult children and other relatives who introduced participants to technology, it makes sense that they would be the ones providing the lessons and education. Third, participants may have been learning from younger generations because they may have a greater knowledge of technology, having grown up in the information age. Fourth, older adults might choose to learn from younger family members as they use less confusing terminology (compared to user manuals or classes) and they are comfortable enough to ask questions. Many older adults in the present study used technology comfortably and were among the early adopters of computers and technology. The role younger generations play in guiding and motivating older adults to use technology may contribute to family cohesion and strengthen relationships. This supports the notion of the “change in family roles” put forward by Taipale [28], who highlighted the variance of perception between Italian and Slovenian contexts.

Nearly all participants reported using a computer at their own home, but other locations such as an adult child’s home were also identified. Studies have shown that, among older adults who use computers, a majority do so in the comfort of their own home, although computers are also used in public locations such as at work, in a library, or at a friend’s/family member’s home [14,37,53,70]. Computers might be used at an adult child’s home because this is where the learning and introduction to technology take place. However, this pattern of usage could also be indicative of locational convenience, access to computers, privacy issues, what the computer is being used for, or another combination of variables. These preliminary findings point to the importance of investigating further how these intergenerational factors influence the location of technology use. 

Even though participants highlighted the many benefits and uses of technology, some participants remarked on the drawbacks and risks of living in the digital age. The finding that older adults often chose to use computers for leisure to share information and communicate, whereas cell phones were often used to appease worried children, suggests both positive and negative associations of technology. For instance, surveillance and privacy issues, along with digital crime, are risks of using certain technologies [71]. Despite the existence of privacy legislation, there exist privacy threats with the use of technology, such as the tracking of personal information, profiling, and privacy-violating interactions [72]. Despite voiced concern over privacy issues, participants continued to use technologies because of the benefits, such as bridging geographical distances to communicate with younger family members. As such, it seems the rewards outweigh the risk for older adults to use technology. Nonetheless, the acknowledgment of such risks by participants draws attention to the importance of providing clear education communicated in lay language on how to safely use technology. 

This research specifically addressed intergenerational elements of technology use among individuals in both rural and urban areas in two countries. Research often overlooks social elements of technology use, viewing technology engagement as a solo activity. A strength of this study is the combination of an in-depth online survey and focus groups, which allowed for a deeper understanding of the topics being studied. Upon further validation, the survey could be used in future studies as a standard measure of technology use, social media habits and behaviour, information sharing, and privacy issues. Given the exploratory nature of the study, a small sample was acceptable as the aim was for each site to recruit 10 participants. Although our sample sizes enabled us to reach saturation of information, a larger sample is needed to confirm our findings. Differences in the recruitment methods across sites may have contributed to the difficulties of achieving the targeted number of participants. Future studies should recruit participants who use and who do not use technology to compare and contrast their behaviours and identify further barriers to and enablers of technology use in later life. Further investigations may extend this work to examine the intersection of technology and intergenerational relationships among older adults who are aging without family to expand the understanding of the roles that peers, friends, or even siblings play in comparison to that of adult children [73,74].

## 5. Conclusions

At a time when technology development and population aging research are prevalent, it is vital to capitalize on opportunities to learn about how technology can be used and deployed to increase social connectedness, improve the quality of life of older adults, and support aging in place. With rapid technological developments occurring, there are great opportunities to expand the understanding of gerontechnology and human–computer interaction from a multi-disciplinary standpoint. Technology has the potential to play an integral role in ensuring all attributes complement each other and keep knowledge up to date. Many participants used technology to maintain social connectedness with younger family members who were geographically dispersed. The findings from this study provide insight into the strengths and opportunities that technologies provide to older adults. Understanding how intergenerational relationships impact technology use in later life can inform further research and technological and social practices.

## Figures and Tables

**Table 1:** Characteristics and overview of technology use for all participants (*n* = 37).

Characteristics	Total Population 100% (*n* = 37)	Canada 47.2% (*n* = 16)	United Kingdom 56.8% (*n* = 21)
Mean age, years ±SD	77.4 ±6.4	79.3 ±5.9	75.9 ±6.6
Age range in years	67–89	70–89	67–89
Gender			
Female	67.6 (25)	87.5 (14)	52.4 (11)
Male	32.4 (12)	12.5 (2)	47.6 (10)
Have used a computer	97.3 (36)	93.8 (15)	100.0 (21)
Own a computer	89.2 (33)	93.8 (15)	85.7 (18)
Own a cell phone	70.3 (26)	50.0 (8)	85.7 (18)
Technology use/ownership			
Play video games	56.3 (18)	50.0 (8)	47.6 (10)
Own a digital/video game console	8.1 (3)	6.3 (1)	9.5 (2)
Have the internet at home	89.2 (33)	81.3 (13)	95.2 (20)
Use social media sites	35.1 (13)	50.0 (8)	23.8 (5)
Email	78.4 (29)	81.3 (13)	72.6 (16)

Note: SD denotes standard deviation; unless noted otherwise, responses are presented as percentages of total responses with the numbers of participants in brackets.

**Table 2:** Characteristics of social media use among participants who reported using social media (*n* = 20/37).

Characteristics	Participants Who Use Social Media 54.1% (*n* = 20/37)	Canada 62.5% (*n* = 10/16)	United Kingdom 47.6% (*n* = 10/21)
Person who introduced participant to social media			
Spouse/partner	5.0 (1)	10.0 (1)	-
Adult child	20.0 (4)	20.0 (2)	20.0 (2)
Friend	40.0 (7)	20.0 (2)	80.0 (5)
Relative	20.0 (4)	40.0 (4)	-
Other	5.0 (1)	10.0 (1)	-
Reasons to use social media			
Connect with friends	70.0 (14)	70.0 (7)	70.0 (7)
Connect with children/grandchildren	70.0 (14)	80.0 (8)	60.0 (6)
Share information with friends/family	50.0 (10)	60.0 (6)	40.0 (4)
Share photos with friends/family	60.0 (12)	60.0 (6)	60.0 (6)
Organize events	20.0 (4)	20.0 (2)	20.0 (2)
Participate in events/groups	15.0 (3)	20.0 (2)	10.0 (1)
Keep up to date with news	40.0 (8)	20.0 (2)	60.0 (6)
Express opinions/views	15.0 (3)	10.0 (1)	20.0 (2)

Note: Responses are presented as percentage of total responses with the number of participants in brackets.

## Supplementary Materials

The following are available online at https://www.mdpi.com/1660-4601/17/16/5711/s1.
