# Supplementary material for: Intergenerational Effects on the Impacts of Technology Use in Later Life: Insights from an International, Multi-Site Study
Source: Int J Environ Res Public Health. 2020 Aug 7;17(16):5711. doi: 10.3390/ijerph17165711 (PMC7459619; doi:10.3390/ijerph17165711)
Supplement: Supplementary file 1 [file ijerph-17-05711-s001.pdf]

Supplemental File 1

Semi-Structured Interview Guide for the Technology In Later Life (TILL) Project

The first questions aim to better understand the types of technology participants use in general and get the conversation started.

1. **Can you tell me about the technologies you use?**
   - Probe for details to describe the technology use
     - i Please describe the type of technology.
     - ii What is the purpose of using this technology?
     - iii How long has you been using the technology?
     - iv What is the frequency of technology use?

The next set of questions aim to better understand the types of technology users, their practices, and preferences. The main question are numbered and bolded with suggested probes to be used where needed as bullet points below

2. Do participants use the Internet? If so, can you tell me about how you use the internet? • How long have you been using the Internet?

- What is the purpose of using the Internet?
- Please describe if and how you share information on the Internet.

3. Do participants engage with video games? If so, can you tell more about how you engage with video games? • What video games are you familiar with and why?

- What are your perceptions of those who play video games?
- What are the benefits of playing games?
- What are the drawbacks or challenges of playing games?

4. Do you record information about yourself using technology? (Can provide examples such as medication use, physical activities, financials etc. if needed) • How long have you been recording your information?

- How would you describe the frequency of your recording? Are you a dedicated recorder?
- What is your purpose for recording information?
- What method of recording information do you use? (traditional – pen/paper; technology – using a digital device, computer etc.)

5. How important is protecting and safeguarding the information you collect or share using technology?

- Do you share your information with anyone? If so, whom do you share information with?
- Does this make you think about protecting your information? If participant answers yes, encourage them to elaborate.

6. What are the advantages of collecting your information using technology? • What have you learned?

- How has it changed what you do?
- Are there any disadvantages to colleting your information?
- What could be done better?

7. If you were to design a device to collect your information what would it look like? • What functions would the device perform?

- What function would not be included?
- What would the information collected be used for?

8. How quickly do you embrace new technologies?

• What kind of things/devices/technologies have you used in the past?

• How do you feel about new technologies that are emerging? i. Consider Robots
ii. Consider more intuitive software/technology through speech & touch

• Do you get help from family and friend on how to use new technologies? i. Which family and friends do you connect with to use new technologies?
ii. Can you share an example?

9. Do you have any additional comments you would like to add?
